# Experimental determination and thermodynamic optimization of the LiF-NdF_3_ system

**DOI:** 10.1039/d3ra03003b

**Published:** 2023-08-11

**Authors:** ChunFa Liao, ZanHui Fu, LiangHua Que, Hao Tang, Xu Wang

**Affiliations:** a Faculty of Materials Metallurgy and Chemistry, Jiangxi University of Science and Technology Ganzhou 341000 China sci_rech@163.com; b National Rare Earth Functional Materials Innovation Center Ganzhou 341000 China

## Abstract

Neodymium is mainly obtained by electrolysis of a molten LiF-NdF_3_-Nd_2_O_3_ system. LiF-NdF_3_ is a basic system, and the phase diagram of this system provides important information in the production of electrolytic neodymium. An accurate LiF-NdF_3_ binary phase diagram helps in the selection of the appropriate molten salt component in production and optimizing the production process, which is of great significance to improve the electrolysis efficiency and reduce the production cost. To obtain an accurate phase diagram of the LiF-NdF_3_ binary system, liquidus and solidus temperatures were experimentally determined in the LiF-NdF_3_ binary system by differential scanning calorimetry. The experimental results were used to construct the phase diagram and develop a new database for the LiF-NdF_3_ system using the FactSage software. The sub-regular solution model was used to describe the excess Gibbs free energy of the liquid phase, and the thermodynamic optimization calculation was carried out for the binary system. The binary interaction coefficients ^0^*L* = −39 966 + 17.68 *T*, ^1^*L* = −7667 + 26.1 *T*, and ^2^*L* = −6000 were used to describe the system's excess Gibbs free energy. The results show that the eutectic point of the system is 68.4% LiF-31.6% NdF_3_ at 731.5 °C. The effects of industrial and high purity NdF_3_ and the presence of Nd_2_O_3_ on the liquidus temperature of the LiF-NdF_3_ system were also investigated, high liquidus temperatures have been observed in tests using industrial NdF_3_ and NdF_3_ feedstock that contains a specific quantity of Nd_2_O_3_.

## Abstract 2

The thermal data of the LiF-NdF_3_ binary system were optimized, and the LiF-NdF_3_ binary phase diagram was constructed by combining experimental measurement with phase diagram calculation technology.

## Graphical abstract

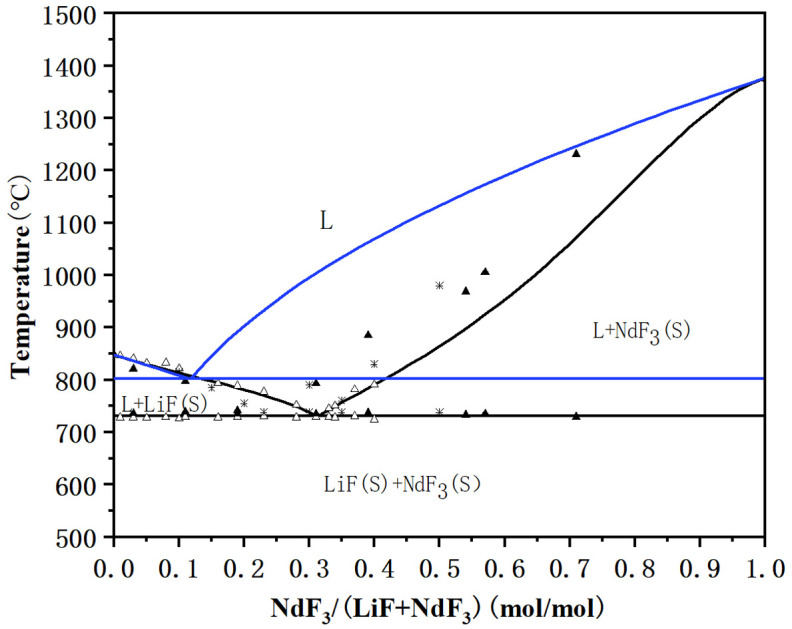

## 1. Introduction

Neodymium is a metal that is widely used in the field of key rare-earth functional materials, such as NdFeB magnetic materials, rare-earth alloys, and rare-earth hydrogen-storage materials.^1,2^ The main preparation method is molten salt electrolysis, and the electrolytic system mainly includes chloride and fluoride salt systems. The fluorine salt system is currently used for industrial production. In the molten salt electrolysis process, rare-earth fluoride is the electrolyte, and rare-earth oxide is electrolytic raw material. Currently, REF_3_-LiF and REF_3_-LiF-BaF_2_ electrolyte systems are commonly used.^3,4^ The solubility and eutectic temperature of rare-earth oxides in the REF_3_-LiF molten salt system are the key factors affecting electrolytic neodymium's production process.^5^ Research into phase diagrams can provide many useful pieces of information for developing new materials: Fedrov *et al.*^6^ reviewed lithium rare-earth fluorides as photonic materials, citing many phase diagrams that can provide theoretical guidance for optimizing production processes. The construction methods of phase diagrams mainly include the experimental determination method^7^ and the CALPHAD phase diagram calculation method.^8,9^ A large amount of experimental data is required to determine phase diagrams, and the accuracy of the phase diagram is greatly affected by the purity of the raw materials, as well as the accuracy of the analysis and experimental operation, which means that it has the disadvantages of a large workload and requires a high level of manpower, large quantities of materials, and substantial financial resources. Phase diagrams are difficult to determine through experiments only, and thermodynamic calculations of phase diagrams are often limited by a lack of accurate thermodynamic data. Experimental and thermodynamic data on the LiF-NdF_3_ system's binary phase diagrams are lacking, with Thoma data and Xue data among the few experimental data found so far. Marcelle Gaune-Escard *et al.*^10^ analyzed the enthalpy of mixing NdF_3_-MF (M = Li, Na, K) binary systems data using the Hoch–Arpshofen solution model, and with the development of CALPHAD phase diagram calculation technology, the construction of phase diagrams by combining experimental determination with phase diagram calculation has been widely adopted by scientific and technological workers.^11,12^

Thoma *et al.*^13^ studied the phase diagram of LiF-NdF_3_ by thermal analysis experiments; however, the raw NdF_3_ material used in the experiments was prepared from the transformation of rare-earth oxide, which had a high level of rare-earth oxide (300 ppm). Nd_2_O_3_ in the raw material had considerable influence on the measurement result of liquidus temperatures. In terms of phase diagram calculation, Van der Meer *et al.*^14^ calculated the NdF_3_-LiF binary phase diagram by using the CALPHAD method and ChemSage software. They used the Redlich–Kister polynomial to describe the excess free energy of the liquid phase based on the literature experimental data.^13^ The calculated phase diagram is consistent with the experimental phase diagram in literature.^13^ M. Berkani *et al.*^15^ calculated the LiF-NdF_3_ binary phase diagram by thermal analysis and the Hoch–Arpshofen model. A. Abbasalizadeh *et al.*^16^ conducted experimental research and thermodynamic modeling on the LiF-NdF_3_-DyF_3_ ternary system, as well as Gibbs free energy modeling of the LiF-NdF_3_ and LiF-DyF_3_ systems by using the literature data.^13^ The phase diagram of the LiF-NdF_3_ binary system was calculated by the CALPHAD method. Xue *et al.* used DTA analysis to measure the liquidus temperature of the NdF_3_-LiF binary system at various compositions,^17^ drew the experimental phase diagram according to the results of the DTA, and finally optimized the LiF-NdF_3_ binary phase diagram according to CALPHAD calculation; however, the experimental materials used were all industrial grade with impurities. The peak temperature of the DTA curve was considered the result of liquidus temperature, and the optimized phase diagram of the LiF-NdF_3_ binary system obtained by this method still requires further discussion. Johnathon C. Ard *et al.*^18^ assessed and then reassessed the thermodynamics of 30 pseudo-binary and ternary salt systems, assessed the LiF-NdF_3_ binary phase diagram by modified quasi-chemical model in the quadruplet approximation with the data from literature,^13^ and determined relations for the liquid interaction parameters, and the phase diagram was in good agreement with literature.^13^ Regarding for the bivariate phase diagram of LiF-NdF_3_ that was previously described, the experimental method measured the phase diagrams,^13,17^ however, the raw material used in the ref. 17 experiment is industrial level, and its phase diagram is only suitable for guiding production. The other is a phase diagram that was calculated using experimental data found in literature.^13^ Additionally, the experimental NdF_3_ material utilized in ref. 13 is not high pure, which will significantly affect the experiment's results. For the purpose of obtaining more accurate phase diagram and thermodynamic data, this research combines the phase diagram calculation technique with higher purity raw materials to measure the solidus and liquidus temperature. Thermodynamic data consistent with the phase diagram can provide basic data for the study of the LiF-NdF_3_-Nd_2_O_3_ ternary phase diagram, which has important practical significance and economic value for optimizing the rare earth neodymium production process, improving current efficiency and reducing production costs. Therefore, differential scanning calorimetry was used to investigate the liquidus and solidus temperatures of the LiF-NdF_3_ system with different components. FactSage software was employed to calculate and optimize the phase diagram of the LiF-NdF_3_ molten salt system. The thermodynamic parameters of the system were optimized, and an accurate LiF-NdF_3_ phase diagram was constructed by combining the experiment with the CALPHAD method.

## 2. Experimental methods

### 2.1. Thermal analysis experiment

Raw materials for thermal analysis experiment: LiF, NdF_3_ and Nd_2_O_3_with purity ≥ 99.99% were obtained from Shanghai Macklin Biochemical Technology Co., Ltd. Industrial grade NdF_3_ obtained from Nanjing Chemical Reagent Co., Ltd.

#### 2.1.1. Instrument

Differential scanning calorimeter (model STA449 F5) was made by Germany NETZSCH Instrument Manufacturing Co., Ltd.

#### 2.1.2. Sample preparation

LiF and NdF_3_ were dried at 150 °C for 24 hours prior to preparation to remove possible moisture in the reagent. LiF-NdF_3_ mixtures of different mass ratios were prepared and ground repeatedly in an agate mortar for half an hour so that LiF and NdF_3_ could be thoroughly mixed, and the preparation process was carried out in a glove box filled with nitrogen gas.

#### 2.1.3. Experiment condition

**Fig. 1:**
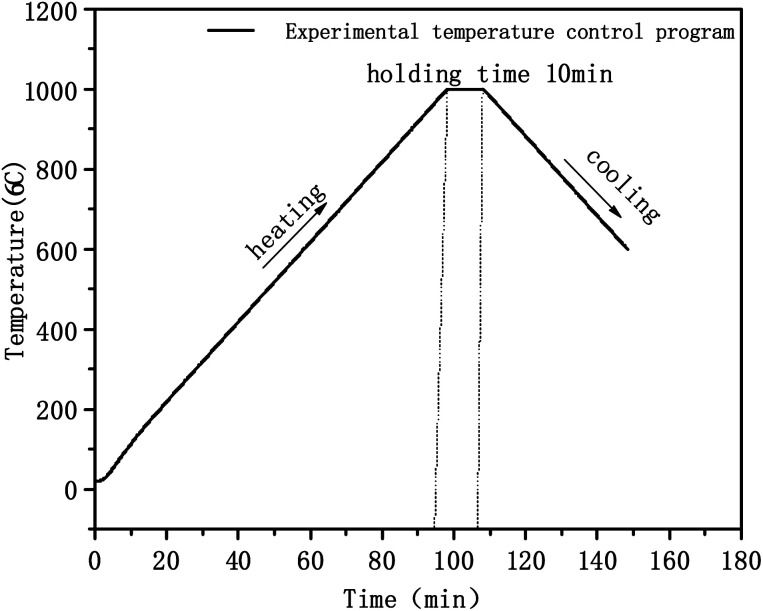
Experimental temperature control procedure.

Thermal analysis experiments were carried out in a nitrogen atmosphere using a platinum crucible. The nitrogen flow velocity was 20 ml min^−1^, the sample weight was 10–20 mg, the highest test temperature was 1000 °C, the lowest test temperature was 600 °C, and the heating and cooling rates were 10 °C min^−1^. The experimental temperature control program is shown in Fig. 1.

### 2.2. Phase diagram calculation

Based on the experimental phase diagram, the Gibbs freedom of the LiF-NdF_3_ system was calculated and optimized using the compound, solution, equilib, optisage, and phase diagram module of FactSage8.1 software. A new compound database and liquid phase database were created in the compound and solution modules, respectively. The interaction coefficient ^*i*^*L* was optimized by the Bayesian optimization algorithm based on the probability model and constantly adjusted until the optimized phase diagram was in good agreement with the experimental phase diagram.^19^ The optimized thermodynamic data and optimized phase diagram of the system were obtained.

## 3. Results and discussion

### 3.1. Experimental results and discussion of thermal analysis

#### 3.1.1. Experimental principle of thermal analysis

**Fig. 2:**
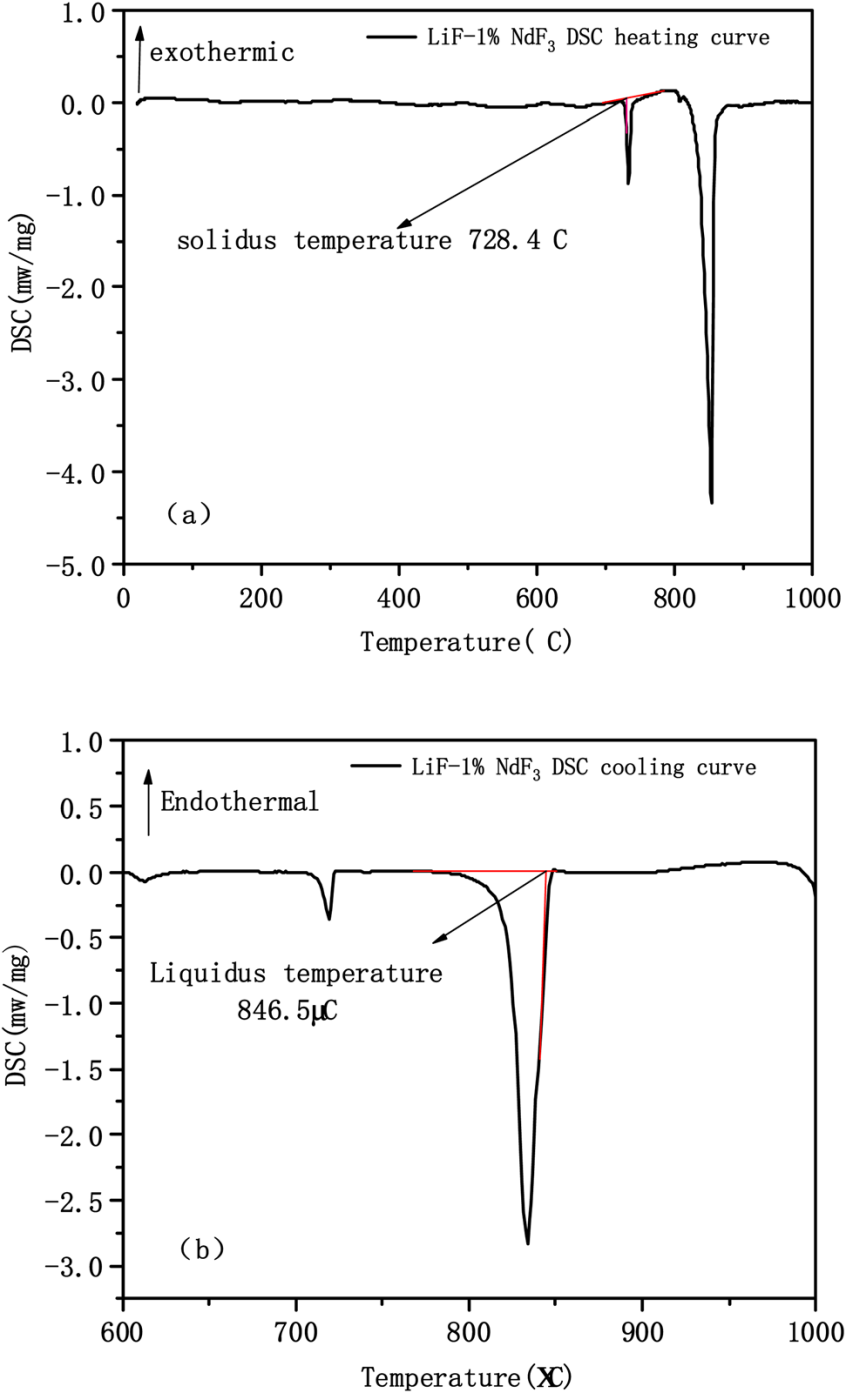
Schematic of solidus and liquidus temperatures analysed by DSC curve: (a) LiF-1% NdF_3_ DSC heating curve; (b) LiF-1% NdF_3_ DSC cooling curve.

The sample exhibited evident endothermic or exothermic phenomena during the phase transformation in the heating and cooling processes. The initial temperature of the endothermic peak is extrapolated from the heating DSC curve as the solidus temperature, and the temperature of the intersection between the tangent line of the maximum slope of the first exothermic peak of the cooling DSC curve and the baseline is considered the liquidus temperature.^5,20^ The reason we obtain the solidus and liquidus temperatures from the different DSC curves is that we observed that the cooling curve is prone to deviation when determining the solidus temperature, which may result in a larger error when the experimental temperature is higher; therefore, we recommend using the heating and cooling curves to determine the solidus and liquidus temperatures, respectively.^21^Fig. 2(a) and (b) show the DSC heating and cooling curves of the binary system LiF-1% NdF_3_, respectively, according to the method. The LiF-NdF_3_ system's liquidus and solidus temperatures are 846.5 °C and 728.4 °C, respectively. Similarly, the liquidus and solidus temperatures of other component samples can be determined.

#### 3.1.2. Experimental results of thermal analysis

**Fig. 3:**
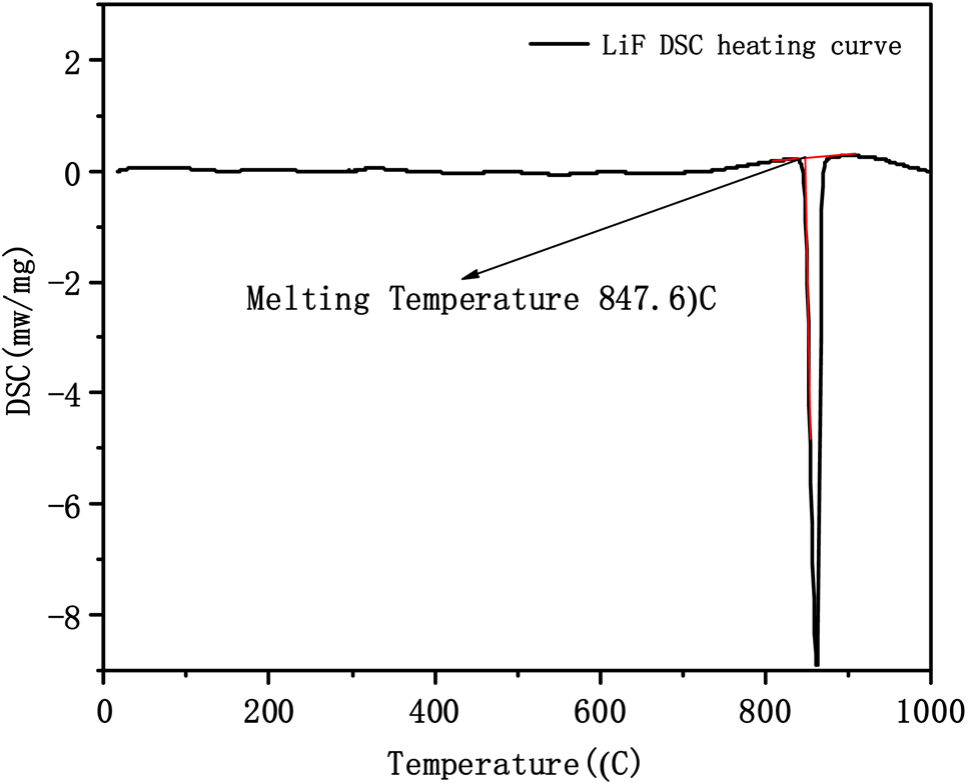
LiF DSC heating curve.

Reports indicate that the melting temperature of LiF is 847.5 °C,^22,23^ while that of NdF_3_ is 1374 °C,^22^ however, some reports show that the melting temperature of NdF_3_ is 1413 °C.^23^ Therefore, the melting temperature of pure-substance LiF was measured with a calibrated thermal analyzer, and the tangent method was used to analyze the DSC heating curve of LiF.^24^Fig. 3 shows that the measurement result is 847.6 °C. The melting temperature of LiF is determined at 847.5 °C, considering the influence of experimental errors. At the same time, the reliability of analyzing the solidus temperature of the molten salt system by the DSC heating curve with tangent method is verified. The melting temperature of NdF_3_ at 1374 °C,^22^ which was reported in the same literature as LiF, was adopted.

Table 1 shows the liquidus and solidus temperatures of the LiF-NdF_3_ molten salt system with different mass fractions tested experimentally. In accordance with the liquidus and solidus temperatures of the LiF-NdF_3_ system measured by thermal analysis, the experimental results show that, when the molar concentration of NdF_3_ is less than 31%, as the mole fraction of NdF_3_ in the system increases, the liquidus temperature of the LiF-NdF_3_ molten salt system gradually decreases. In addition, when the molar concentration of NdF_3_ is greater than 31%, as the increase in the mole fraction of NdF_3_ in the system increases, the liquidus temperature of the LiF-NdF_3_ system gradually increases. This finding shows that LiF can significantly reduce the liquidus temperature of the molten salt system, and with the increase in the mole fraction of NdF_3_, the liquidus temperature of the LiF-NdF_3_ system gradually increases mainly due to the high melting temperature of NdF_3_. The samples of different components have the same solidus temperature, which also indicates that the LiF-NdF_3_ system is a low eutectic system without the formation of intermediate compounds, which is consistent with the results of previous studies.

**Table 1:** DSC curve analysis results of different compositions of LiF-NdF_3_ system

No.	LiF mol%	NdF_3_ mol%	Liquidus temperature (°C)	Solidus temperature (°C)	Ref.
1	100	0	847.5		22
2	99	1	846.5	728.4	
3	97	3	841.1	728.3	
4	95	5	832.6	727.1	
5	92	8	832.7	728.8	
6	90	10	821.8	726.1	
7	89	11	798.3	729.2	
8	84	16	793.6	728.2	
9	81	19	788.8	729.3	
10	77	23	776.9	729.7	
11	72	28	752.2	728.3	
12	69	31	734.6	729.1	
13	67	33	745.4	729.4	
14	66	34	751.1	728.4	
15	63	37	782.0	730.6	
16	60	40	790.5	724.4	
15	0	100	1374		22

Table 2 shows the regression equation, indicating that the intersection of the two fitted lines is the eutectic point of the LiF-NdF_3_ system. After calculation, the eutectic temperature of the system is 736.6 °C, and the eutectic components are divided into 68.62% LiF-31.38% NdF_3_.

**Table 2:** Regression equation of liquidus temperature (°C) for LiF-NdF_3_ binary system^a^

Range (mol%)	Fitted equation
*x*_NdF_3__ > 31%	*Y* = 577.59 + 374.2858 × *C* + 422.1014 × *C*^2^
*x*_NdF_3__ ≤ 31%	*Y* = 848.59 − 287.4729 × *C* − 220.8428 × *C*^2^

a Note: *Y*—liquidus temperature (°C); *C*—mole fraction of NdF_3_.

### 3.2. LiF-NdF_3_ phase diagram calculation and thermodynamic optimization

#### 3.2.1. Theoretical basis of phase diagram calculation

The first step to describe the phases of the LiF-NdF_3_ molten salt system is to define the Gibbs free energy functions for all the compounds and mixtures in the system. In most instances, the excess Gibbs free energy function is unknown. Therefore, thermodynamic evaluation is required to determine the excess Gibbs free energy. The Gibbs free energy equation of the compound is a function of enthalpy and entropy at the standard temperature state (298.15 K), as follows:

1 *G*(*T*) = *H*(*T*) − *S*(*T*)*T*

2
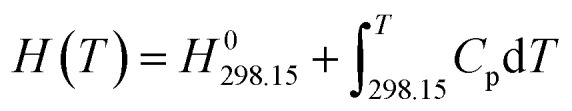
3
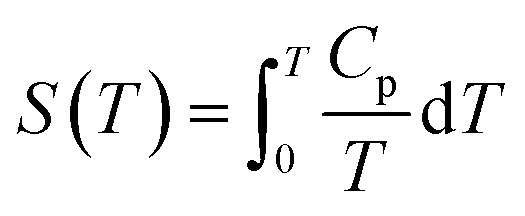


*C*_p_ function can be obtained by the fitting group experimental data:

4 *C*_p_ = *a* + *bT* + *cT*^−2^

In the LiF-NdF_3_ binary molten salt system, the pure components are LiF and NdF_3_, the crystal structures are shown in Table 3, and the Gibbs free energy for pure components can be expressed as a function of temperature, as follows:5
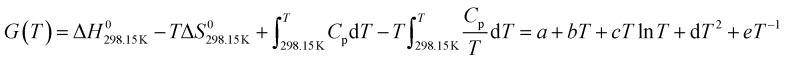
where Δ*H*298.150 and Δ*S*298.150 are the standard enthalpy and standard entropy of the standard state (1 bar) at 298.15 K, respectively; *C*_p_ is the isobaric heat capacity of each pure substance; *a*, *b*, *c*, *d*, and *e* are the parameters to be determined; and *T* is the thermodynamic absolute temperature, K. Specific data are shown in Table 4, and the related thermodynamic parameters of pure components are obtained from the FactSage database.^25^

**Table 3:** Phase and crystal structure adopted for thermodynamic calculation of the LiF-NdF_3_ system

Phase	Space groups	Crystal structure	Descriptive model
Liquid	—	—	[LiF, NdF_3_]
LiF	*Fm*3*m*	Cubic	LiF
NdF_3_	*P*6*c*2	Hexagonal	NdF_3_

**Table 4:** Thermodynamic parameters of LiF and NdF_3_ pure substances

Phase	Δ*H*_298.15_ kJ mol^−1^	*S*_298.15_ J mol^−1^ K^−1^	Temperature, K	*C*_p_ J mol^−1^ K^−1^
LiF (solid)	−616.931	35.65	298.15–1121.3	*C*_p_ = 42.69 + 14.41 × 10^−3^*T* − 5.30 × 10^5^ × *T*^−2^
LiF (liquid)	−594.58	43.00	1121.3–1900	64.183
NdF_3_ (solid)	−1679.458	120.792	298.15–1650	*C*_p_ = 92.697 + 23.43 × 10^−3^ × *T* − 6.456 × 10^5^ × *T*^−2^
NdF_3_ (liquid)	−1607.88	340.77	1650–1800	*C*_p_ = 184.473 − 4.435 × 10^−3^*T* − 119.244 ×10^5^ × *T*^−2^

The most commonly used thermodynamic models for phase diagram calculation are the ideal solution, regular solution, compound energy, modified association models, as well as the modified quasi-chemical model in the quadruplet approximation.^26^ The regular solution model^27^ was proposed by Hildebrand in 1929, assuming the actual melt to be a replacement melt, and it is well simulated in some simple melt systems. The LiF-LuF_3_ molten salt systems (Lu = La, Ce, Pr, Nd, Sm, U and Pu) are all phase diagram systems with a low eutectic point without forming an intermediate, therefore, the thermodynamic LiF-NdF_3_ molten salt system can be described by the sub-regular solution model. Moreover, in the literature,^15–17^ the regular solution model was used to calculate the phase diagram of the LiF-NdF_3_ binary system.

For the liquid phase of the LiF-NdF_3_ binary system, the sub-regular solution model was used to describe the Gibbs free energy in the liquid phase. The Gibbs free energy is composed of pure-component mechanical mixing to Gibbs free energy, an ideal mixing contribution to Gibbs free energy, and excess Gibbs free energy.^28–30^ The expression is as follows:

6 *G*^liquid^ = ref*G*^liquid^_m_ + ^id^*G*^liquid^_m_ + ^ex^*G*^liquid^_m_

7
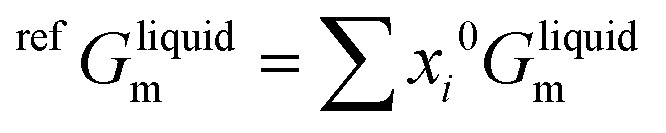
8
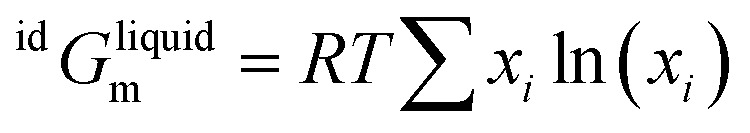
where ^ref^*G*_m_^liquid^ is the contribution of pure-component mechanical mixing to Gibbs free energy, ^id^*G*_m_^liquid^ is the contribution of ideal mixing entropy to Gibbs free energy, and ^ex^*G*_m_^liquid^ is the part of the non-ideal mixture, that is, the contribution of the excess Gibbs free energy. The excess Gibbs free energy was expressed by the Redlich–Kister polynomial.^31^ When calculating the LiF-NdF_3_ binary phase diagram, the thermodynamic model can be described as follows:9
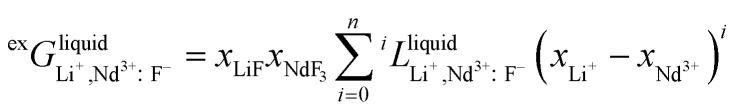
where the parameter ^*i*^*L* has a linear relationship with temperature and can be described as follows:

10 ^*i*^*L* = *a* + *bT*

where *x*_LiF_ and *x*_NdF_3__ are the mole fractions of LiF and NdF_3_, respectively, and ^*i*^*L* is the binary interaction coefficient of order *i*. When *i* = 0, ^ex^*G*_m_^liquid^ becomes a regular solution model, and when *i* = 1, ^ex^*G*_m_^liquid^ becomes a sub-regular solution model. In general, the value of *i* is not greater than 2, and *a* and *b* are parameters that should be optimized.

#### 3.2.2. Phase diagram calculation and thermodynamic optimization

**Fig. 4:**
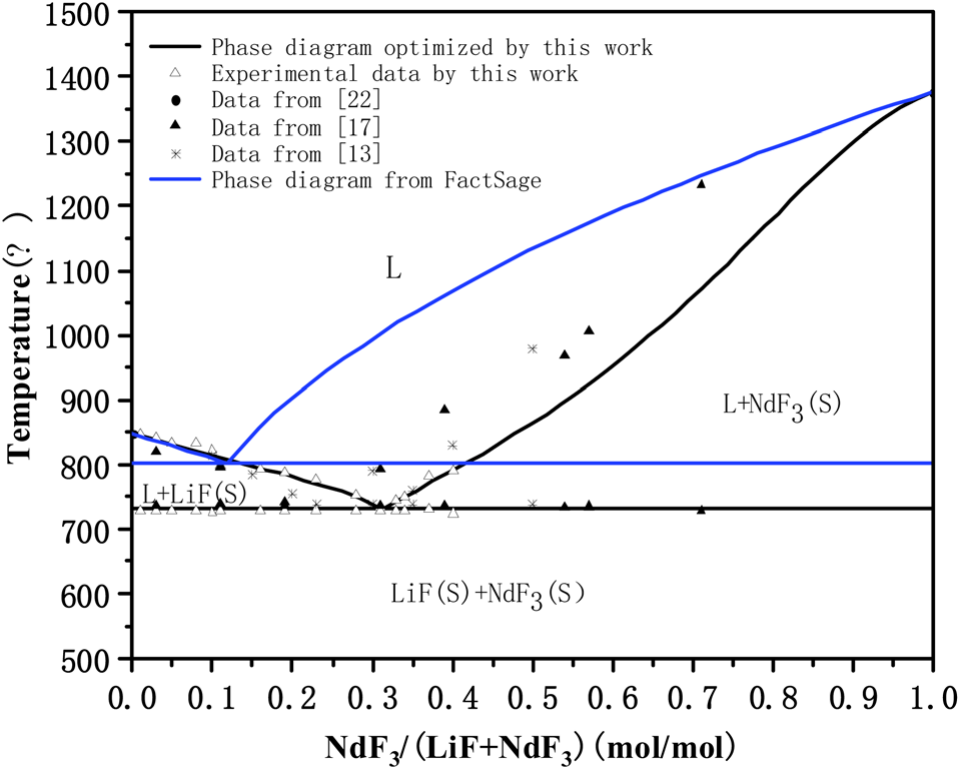
Comparison between optimized LiF-NdF_3_ binary phase diagram and experimental data. * The blue line was calculated directly by FactSage software, which was not optimized.

Table 5 shows the optimized system thermodynamic data and thermodynamic interaction parameters, while Fig. 4 shows the optimized phase diagram, both according to the phase diagram calculation method described in Section 2.2.

**Table 5:** Thermodynamic optimization parameters of LiF-NdF_3_ binary molten salt system

System	Phase	Gibbs free energy parameter (J mol^−1^)
LiF-NdF_3_	LiF	*S*: *G* = −628 236.010 + 258.443 *T* − 42.689*T* ln *T* − 0.009 *T*^2^ + 265 056.4 *T*^−1^ (298.15–1121.3 K)
		L: *G* = −613 717.638 + 386.871 *T* − 64.183*T* ln *T* (1121.3–2000 K)
	NdF_3_	S: *G* = −1 686 591.566 + 386.191 *T* − 74.977*T* ln *T* − 0.0183 *T*^2^ − 1 014 620 *T*^−1^ (298.15–1650 K)
		L: *G* = −1 765 032.569 + 1208.505 *T* − 184.473*T* ln *T* + 0.002 *T*^2^ + 5 962 200 *T*^−1^ (1650 − 1900 K)
	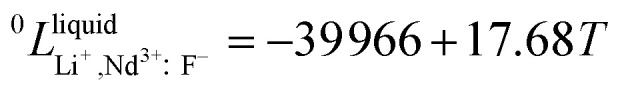	
	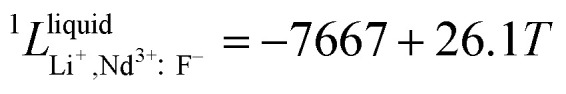	
	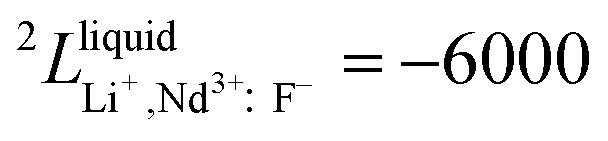	

Under the system's optimized thermodynamic data, the liquidus temperatures of each experimental component were calculated using the Equilib module in FactSage, with the maximum, minimum, and average deviations being 12.54 °C, −0.53 °C, and 3.28 °C, respectively. The eutectic temperature of the optimized phase diagram is 731.5 °C, and the eutectic point of the system is 68.4% LiF-31.6% NdF_3_. The result is very close to the intersection of the two fitted lines calculated in Section 3.1.2, showing that the calculated phase diagram is in good agreement with the experimental phase diagram. Moreover, describing the Gibbs free energy of the LiF-NdF_3_ molten salt system is feasible by using the sub-regular solution model. The calculated phase diagram is accurate and reliable.

Fig. 4 shows that the phase diagram obtained in this work (calculated directly by FactSage software (the blue line)) and the phase diagrams in the ref. 13 and 17 are all simple binary phase diagrams formed without intermediates. The liquidus trend is the same, but there are some differences. The eutectic point position and liquidus temperature at the low LiF side of the system are different. We also noticed that the obtained phase diagram was different from the literature,^17^ although the trend in the temperature of the liquids is consistent, and the composition of eutectics in this paper is 68.4% LiF-31.6% NdF_3_ and 77% LiF-23% NdF_3_ in literature.^17^ The difference might be caused by the equipment being tested, the purity of the raw materials, or other factors. At the same time, the final result may be slightly different because the phase diagram is optimized by different thermodynamic models. The phase diagram that calculated by FactSage directly is the phase diagram without thermodynamic evaluation and optimization, meaning it does not consider the influence of excess Gibbs free energy on the system. However, there is possibility of improvement in the FactSage LiF-NdF_3_ binary system database through the optimized thermodynamic data from the literature.

As is well known that NdF_3_ is primarily based on Nd_2_O_3_ as a raw material for fluorine conversion preparation, that its conversion rate is affected by the production process, that different production processes or requirements for purity are different, that its conversion rate will be different, and that for raw materials of industrial grade, it is typically believed that NdF_3_ purity must be greater than 99.0% to satisfy the requirements. To learn more about how various NdF_3_ raw materials affect the liquidus temperature of the LiF-NdF_3_ binary phase diagram, four comparison experiments were conducted using high-purity NdF_3_ (purity ≥ 99.99%) and industrial NdF_3_ (purity ≥ 99.0%) as the raw materials. Because the liquidus temperature of LiF-NdF_3_ obtained in this paper on the side with high NdF_3_ content is significantly lower than the data obtained in the literature.^17^Table 6 displays the findings of the DSC test. According to the results of the experiments, when NdF_3_ is tested with industrial purity, the liquidus temperature is clearly higher than when NdF_3_ is tested with high purity raw material under the same conditions. The experiments show that when NdF_3_ is tested with industrial purity, for example, the industrial raw material tested this time had a liquidus temperature of 802.4 °C, which is almost identical to the 793.6 °C found in the literature^17^ for the LiF-31% mol NdF_3_ component. This discrepancy may be caused by the different raw material sources, though. The temperature of 734.6 °C that was tested using raw materials of high purity is substantially lower than both of the results.

**Table 6:** Experimental results comparing high purity and industrial NdF_3_

No.	Component	Liquidus temperature with high purity NdF_3_ (°C)	Liquidus temperature with industrial NdF_3_ (°C)
1	LiF-23% mol NdF_3_	776.9	805.0
2	LiF-31% mol NdF_3_	734.6	802.4
3	LiF-37% mol NdF_3_	782.0	797.6
4	LiF-40% mol NdF_3_	790.5	798.2

Simultaneously, to demonstrate the influence of Nd_2_O_3_ impurities on the liquidus temperature of the LiF-NdF_3_ binary phase diagram, the liquidus temperature of the LiF-23% mol NdF_3_ system was 793.8 °C, 781.1 °C, and 803.0 °C after adding the mass fractions of Nd_2_O_3_ of 0.5%, 1.0%, and 2.0%, respectively. The experimental results demonstrate that, in comparison to the system without Nd_2_O_3_, the liquidus temperature of the LiF-NdF_3_ system rises with varied mass fractions of Nd_2_O_3_.

To sum up, on the low NdF_3_ side, the experimental data and optimized liquidus temperature in this work are basically consistent with those in ref. 13 and 17; however, on the low LiF side, the experimental data and optimized liquidus temperature obtained in this work are significantly lower than those in ref. 13 and 17, which may be related to the purity of the NdF_3_ used in the experiments mentioned above, because higher levels of Nd_2_O_3_ or other impurities will affect the liquidus temperature of the system and cause the eutectic point position to shift to the lower NdF_3_ position. This can also explain why the phase diagram in our work is basically consistent with the phase diagram in the literature on the low NdF_3_ side, while there is a larger difference on the low LiF side.

## 4. Conclusions

The thermal properties of the LiF-NdF_3_ binary molten salt system were examined in this study using differential scanning calorimetry. The results acquired from this analysis were utilized a relationship between the component's melting temperature and its NdF_3_ content. When *x*_NdF3_ > 31%, *Y* = 577.59 + 374.2858 × *C* + 422.1014 × *C*^2^, and when *x*_NdF_3__ ≤ 31%, *Y* = 577.59 + 374.2858 × *C* + 422.1014 × *C*^2^. In LiF-NdF_3_-Nd_2_O_3_ molten salt systems, the regression equation can be used as a guide for altering and optimizing the rare-earth neodymium electrolysis process.

The LiF-NdF_3_ binary system's liquidus temperature test findings are significantly influenced by the purity of NdF_3_, and they are likely to deviate greatly if Nd_2_O_3_ or other impurities are present in the raw material. As a result, the experimental phase diagram of the LiF-NdF_3_ binary system was chosen for this paper with a purity of at least 99.99% and is reliable.

The thermodynamic parameters of the basic molten salt LiF-NdF_3_ binary system for the electrolysis of molten salt fluoride and neodymium oxide were optimized using the CALPHAD phase diagram calculation technology, FactSage software, and sub-regular solution model. The thermodynamic data of the LiF-NdF_3_ system were improved, and an accurate LiF-NdF_3_ binary phase diagram was constructed in combination with the experimental data, providing basic data for the construction of the LiF-NdF_3_-Nd_2_O_3_ ternary phase diagram in the next procedure.

More studies are needed on the binary phase diagram of LiF-NdF_3_ that obtained by experimental and CALPHAD methods because the simulation of the system is a very complicated project. In future research, it is necessary to obtain additional experimental data and select more accurate thermodynamic models to optimize phase diagram simulations by comparing the optimization effects of different thermodynamic models. For example, the ref. 18 optimized the phase diagram of LiF-NdF_3_ binary systems by using the modified quasi-chemical model in quadruplet approximation. More thermodynamic data were needed to research the LiF-NdF_3_ binary system. When the experimental data is sufficient, the thermodynamic data is complete and the optimization is more reasonable, the phase diagram of LiF-NdF_3_ will become more and more accurate.

## Conflicts of interest

There are no conflicts to declare.

## Supplementary Material
